# A Biological Retina Inspired Tone Mapping Processor for High-Speed and Energy-Efficient Image Enhancement

**DOI:** 10.3390/s20195600

**Published:** 2020-09-30

**Authors:** Xiaoqiang Xiang, Lili Liu, Luying Que, Conghan Jia, Bo Yan, Yongjie Li, Jinhong Guo, Jun Zhou

**Affiliations:** 1 School of Information and Communication Engineering, University of Electronic Science and Technology of China, Chengdu 611731, Sichuan, China; 201721010408@std.uestc.edu.cn (X.X.); 201752010922@std.uestc.edu.cn (L.L.); 201821010433@std.uestc.edu.cn (L.Q.); 201822010437@std.uestc.edu.cn (C.J.); yanboyu@uestc.edu.cn (B.Y.); guojinhong@uestc.edu.cn (J.G.); 2 School of Life Science and Technology, University of Electronic Science and Technology of China, Chengdu 611731, Sichuan, China; liyj@uestc.edu.cn

**Keywords:** retina, tone mapping processor, high throughput, energy-efficient

## Abstract

In this work, a biological retina inspired tone mapping processor for high-speed and energy-efficient image enhancement has been proposed. To achieve high throughput and high energy efficiency, several hardware design techniques have been proposed, including data partition based parallel processing with S-shape sliding, adjacent frame feature sharing, multi-layer convolution pipelining, and convolution filter compression with zero skipping convolution. Implemented on a Xilinx’s Virtex7 FPGA, the proposed design achieves a high throughput of 189 frames per second for 1024 × 768 RGB images while consuming 819 mW. Compared with several state-of-the-art tone mapping processors, the proposed design shows higher throughput and energy efficiency. It is suitable for high-speed and energy-constrained image enhancement applications.

## 1. Introduction

As one of the major image enhancement approaches, tone mapping has been widely used for recovering the image details in the dark or over-exposure areas in the high dynamic range images, by mapping the original dynamic range to a proper dynamic range. In the past, many tone mapping algorithms have been proposed [1,2,3]. Compared to general tone mapping algorithms, biological retina inspired tone mapping algorithms employ the processing mechanisms of the biological visual system that render the details in the dark areas in a more natural way. In Reference [4], a biological retina inspired tone mapping algorithm is proposed to mimic the retinal visual adaptation and cortical local contrast enhancement in two independent stages. References [5,6,7] proposed revised biological retina inspired tone mapping algorithm with improved performance. Recently, Reference [8] proposed a new biological retina inspired tone mapping algorithm to adaptively adjusts the receptive field (RF) size of horizontal cells (HCs) based on the local brightness, which improves the details in the dark areas. Most of the tone mapping algorithms are implemented on PC using software. This heavily limits the processing speed and makes it unsuitable for portable devices. In recent years, FPGA has been used to implement the tone mapping algorithms. Compared to PC, microcontroller, and DSP, FPGA is pure hardware implementation and the processing can be done in parallel. This significantly improves the processing speed. In the past, different FPGA-based tone mapping processors have been proposed. Reference [1] proposed a tone mapping processor using a local tone mapping algorithm. It adopts different compression levels for each pixel according to the local pixel statistical characteristics. This work achieves real-time processing with high resource utilization. Reference [9] proposed a tone mapping processor using exponent-based tone mapping algorithm [10]. It achieves real-time processing with high image quality, however, it is prone to halos that significantly affect the visual effect. To address this issue, Reference [11] proposed a tone mapping processor using Gaussian filter to reduce effect of the halo. In addition, an automatic key parameter estimation block is used to control the brightness of the tone-mapped images. Reference [12] proposed a biological retina inspired processor employing retina mechanism and histogram equalization. It improves the image details in a more natural way than the conventional methods. Reference [13] proposed a hybrid vision enhancement processor employing optimized tone mapping (OTM) and adaptive gamma correction (AGC) algorithm to achieve improved visual quality. Reference [14] also proposed a hybrid system image enhancement processor. The processor employs contrast limited adaptive histogram equalization (CLAHE) and a spatial filtering based on a bio-inspired retina model to provide enhanced visual quality for visually impaired people. Reference [15] proposed a Tone Mapping processor combining a global compression model and a local contrast enhancement model for each pixel to perform tone mapping. Reference [16] proposed an optimized global tone mapping processor based on the drago operator [17] for high precision image processing. Reference [18] summarize and categorize the state-of-the-art research in tone mapping. Reference [19] reviews the work to date on tone reproduction techniques that includes an investigation into the need for accurate tone reproduction and a discussion of techniques to date. Reference [20] described a subjective experiment attempting to determine users’ preference with respect to these two types of content in two different viewing scenarios—with and without the HDR reference. In addition, deep learning-based tone mapping methods have been proposed recently [21,22,23]. While some of them show better performance than the previous methods, the significantly increased computational complexity makes them unsuitable for energy-constrained image enhancement applications.

The existing tone mapping processors mainly have two issues. Firstly, the processing speed is limited, making it difficult for the designs to be used for high-speed video enhancement applications. Secondly, the energy efficiency of the existing designs is insufficient, making the designs unsuitable for energy-constrained video enhancement applications. In this work, we have proposed a FPGA-based biological retina inspired tone mapping processor. To the best of our knowledge, this is the second FPGA-based biological retina inspired tone mapping processor reported so far (the first one was reported in Reference [12]). Several hardware design techniques have been proposed to achieve high throughput and high energy efficiency for high-speed and energy-constrained image enhancement applications. The experimental results show that the proposed design has better performance and energy efficiency compared with several state-of-the-art tone mapping processors.

## 2. Biological Retina Inspired Tone Mapping Algorithm

This section briefly introduces the biological retina inspired tone mapping algorithm proposed in Reference [8], based on which we designed our tone mapping processor. The algorithm is inspired by the retinal information processing mechanisms of the biological visual system, including the horizontal cells stage and bipolar cells stage.

**Figure 1:**
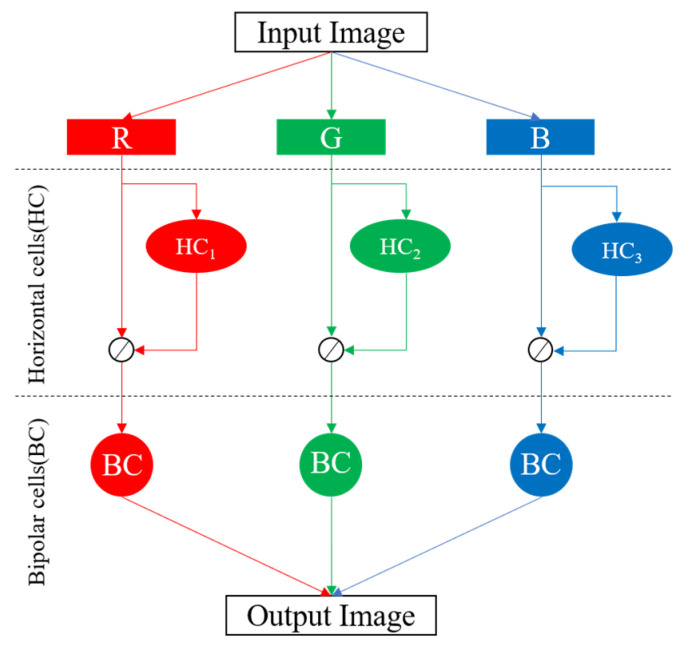
The structure of the algorithm model [8].

One of the major differentiations of this algorithm from other biological retina inspired tone mapping algorithms is the adaptive adjustment of the receptive field size of horizontal cells based on the local brightness, which simulates the dynamic gap junction between the horizontal cells based on the physiological evidence. This enables the brightness of distinct regions to be adjusted into clearly visible ranges while reducing the halo artifacts around the edges of high luminance contrast that are commonly produced by other methods. Figure 1 shows the architecture of the algorithm model.

The horizontal cells stage is used to adjust the brightness of the input image with the 15 × 15 convolution. The 15 × 15 convolution is shown in Equation (1). Where the * represents the convolution operation, $x_{input_{image}}\left(x,y\right)$ represent the input image, $g(x,y;\sigma_{n}\left(x,y\right))$ represents Gaussian convolution filters that is determined by the value (i.e., brightness) range of the central pixel. The $g(x,y;\sigma_{n}\left(x,y\right))$ is shown in Equation (2).

(1) $$x_{horizontal_{cell_{out}}}\left(x,y\right)=x_{input_{image}}\left(x,y\right)*g(x,y;\sigma_{n}\left(x,y\right))$$

(2) $$g(x,y;\sigma_{n}\left(x,y\right))=\frac{1}{2\pi \sigma_{n}^{2}\left(x,y\right)}\;\exp\left(\frac{-\left(x^{2}+y^{2}\right)}{2\sigma_{n}^{2}\left(x,y\right)}\right)$$

For each 15 × 15 convolution, different Gaussian convolution filters are used for each dot multiplication in the convolution according to the value range of central pixel. The selection of the Gaussian convolution filters is determined by Table 1, where m represents mean value and s represents standard deviation and sigma is a parameter defining the max coupling strength of horizontal cells, which is experimentally set to be 1.0 in this work. There are in total 4 Gaussian convolution filters (1, 2, 3, 4), corresponding to the n of $\;g(x,y;\sigma_{n}\left(x,y\right))$ in Equation (1), where n ∈{1, 2, 3, 4}.

The output of the convolution is further processed using Equation (3) before entering the bipolar cells stage. The Equation (2) is to implement a feedback adjustment mechanism to optimize the intermediate results at different stages (i.e., horizontal cells stage and bipolar cells stage) in the processing flow by gain adjustment [8]. In Equation (3), m represents the mean of the input image, $x_{input_{image}}\left(x,y\right)$ and $x_{horizontal_{cell_{out}}}\left(x,y\right)$ represent the input image and the output of the convolution respectively. $y_{BC_{in}}\left(x,y\right)$ represents the input of the bipolar cell stage.

(3) $$y_{BC_{in}}\left(x,y\right)=\;\frac{x_{input_{image}}\left(x,y\right)}{\frac{m}{3}+x_{horizontal_{cell_{out}}}\left(x,y\right)}$$

Bipolar cells are used to enhance the local contrast with a 7 × 7 convolution. It also helps reduce redundant information and improve spatial resolution [8]. After the convolution, the pixels of output image go through an activation function based on Equation (4).

(4) $$y_{out}\left(x,y\right)=\max\left[0,y_{BC_{in}}\left(x,y\right)*x_{Dog}\left(x,y;\sigma_{cen,sur}\right)\right]$$

(5) $$x_{Dog}\left(x,y;\sigma_{cen,sur}\right)=g\left(x,y;\sigma_{cen}\left(x,y\right)\right)-k\cdot g\left(x,y;\sigma_{sur}\left(x,y\right)\right)$$

where the * represents the convolution operation, $x_{Dog}$ represents the 7 × 7 Difference of Gaussian (DOG) convolution filter that is shown in Equation (5). Where k is the relative sensitivity of the repression surround, the k is set to be the 0.3 in this work, $\sigma_{cen}\left(x,y\right)$ and $\sigma_{sur}\left(x,y\right)$ are, respectively, the standard deviations of the Gaussian-shaped receptive fields center and its surround, which are experimentally set to be 0.5 and 1.0, respectively, in this work.

The $y_{out}$ represents the final output of the biological retina inspired tone mapping algorithm. Different from the 15 × 15 convolution which uses 4 filters, the 7 × 7 convolution kernel uses only one filter.

## 3. Proposed Biological Retina Inspired Tone Mapping Processor

**Figure 2:**
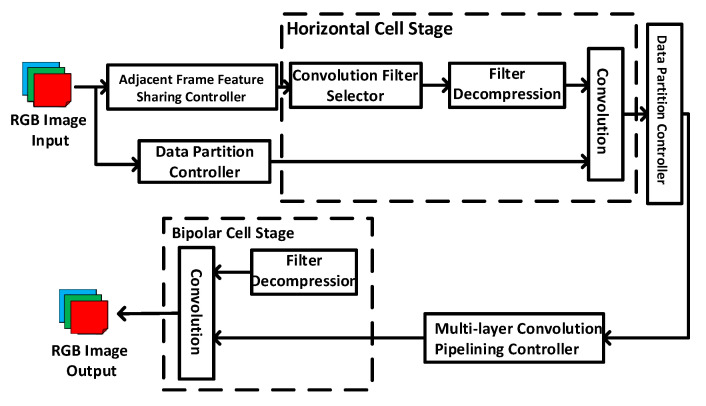
Architecture of proposed biological retina inspired tone mapping processor.

Figure 2 shows the architecture of the proposed biological retina inspired tone mapping processor. The processor implements the horizontal and bipolar cells stages in the biological retina inspired tone mapping algorithm as described in Section 2. In order to achieve high throughput and high energy efficiency, several hardware design techniques have been proposed and implemented in the processor architecture, including data partition-based parallel processing, adjacent frame feature sharing, multi-layer convolution pipelining, and convolution filter compression. The details of these techniques are presented as following.

### 3.1. Data Partition Based Parallel Processing with S-Shape Sliding

**Figure 3:**
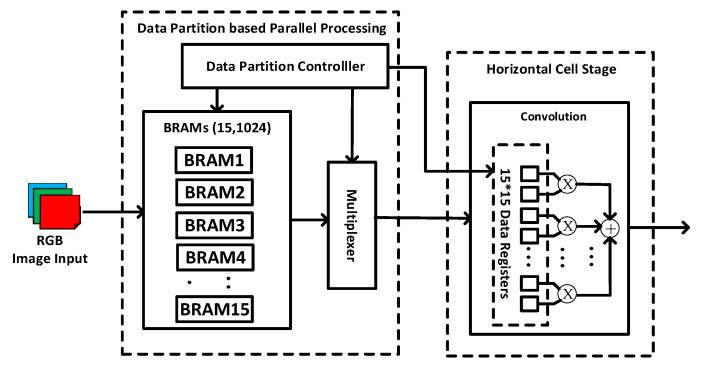
Data partition-based parallel processing with S-shape sliding.

The biological retina inspired tone mapping algorithm involves two convolutions (15 × 15 and 7 × 7). For the 15 × 15 convolution, the input image (1024 × 768) is stored in an on-chip memory (i.e., BRAM in FPGA) and enters the dot multiplication module pixel by pixel. For generating a pixel of output image, 15 × 15 of pixel is needed. When sliding the filter window from left to right, a new column of pixels (1 × 15) needs to be read from BRAM for generating a new output pixel, which requires 15 read cycles. In order to reduce the read time and increase the throughput, the input image is partitioned under a data partition controller and 15 rows of input image are buffered in 15 small BRAMs, each containing 1024 input pixels, as shown in Figure 3. For generating an output pixel, 15 input pixels are read simultaneously from the 15 BRAMs for the dot multiplication and addition. This saves a large number of clock cycles for the generation of each output pixel in the same row. When changing rows, the filter window slides in S shape instead of Z shape so that the input pixels at the end of previous row can be reused. As shown in Figure 5 later, with the S shape sliding, the pixels in the current filter window always overlaps significantly with the pixels in the previous filter window even when changing the row. This allows for data reuse and reduces the number of access to the BRAM, which reduces the processing time and power consumption for data. Here, an issue is that when a row of output image is completed, a new row of input pixels need to be written into one of the 15 BRAMs to start convolution for next row. This causes waiting time of 1024 clock cycles.

In order to save the waiting time, while reading 15 pixels from the 15 BRAMs each time, a new pixel from the 16th row of input image is written into the 1st BRAM. In this way, after a row of output pixels are all generated, the next row of the input pixels is also ready in the 1st BRAM. The convolution of next row can be started immediately without waiting. For performing the dot multiplication, the pixels from different BRAMs are added to the data registers of a 15 × 15 multiplier array through a multiplexer.

The data partition controller dynamically configures the multiplexer and the data registers so that the 15 × 15 pixels are reshaped before dot multiplication. For example, for the first time, the pixels from the 2–15th rows are shifted up and the pixels from the 16th row (stored in 1st BRAM) are moved to the bottom. When writing the 17th row of input pixels, they are written into the 2nd BRAM. The pixels from the 3–15th rows are shifted up and the pixels from the 16th–17th row (stored in 1st–2nd BRAM) are moved to the bottom. The rest may be deduced by analogy until the entire output image is generated. It is noted that zero-padding is involved during the convolution. The same design technique is also applied to 7 × 7 convolution for reducing the processing time and power consumption.

### 3.2. Adjacent Frame Feature Sharing Technique

During the computation of horizontal cells stage, the convolution filter is selected according to the mean value and standard deviation of the input image as described in Section 2. As the calculation of the mean value and standard deviation can only be completed until all the input pixels have been visited, it means that the input pixels have to be stored in a BRAM and read out three times (calculate the mean value first and then standard deviation and then convolution). This consumes significant amount of waiting time and power for data reading.

**Figure 4:**
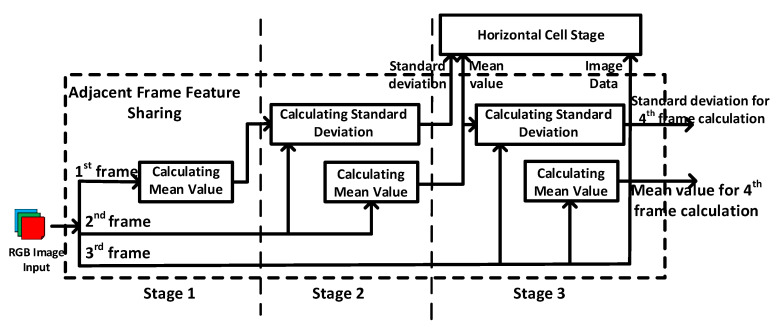
Adjacent frame feature sharing.

In order to reduce the read time and power consumption, we have proposed an adjacent frame feature sharing technique. The basic concept is to leverage the fact that for video processing adjacent input images have similar mean value and standard deviation. As shown in Figure 4, a three-stage processing architecture is designed to realize the concept. The first stage is used to calculate the mean value of the 1st frame. The second stage is used to calculate the standard deviation of the 2nd frame based on the mean value calculated from the first frame.

In the meanwhile, the mean value of the 2nd frame is also calculated at the first stage for later use. The third stage is used to perform the filter selection and convolution for the 3rd frame based on the calculated mean value and standard deviation of the 2nd frame. In the meanwhile, the mean value and standard deviation of the 3rd frame is also calculated at the first and second stages for later use. In this way, the read time and power consumption can be greatly reduced.

### 3.3. Multi-Layer Convolution Pipelining

There are two convolutions involved in the biological retina inspired tone mapping algorithm. One is in the horizontal cells stage (15 × 15) and the other is in the bipolar cells stage (7 × 7). To perform the two convolutions consecutively, a BRAM buffer is needed to store the intermediate data between the two convolutions. However, this will lead to large power consumption for repeated data writing and reading.

**Figure 5:**
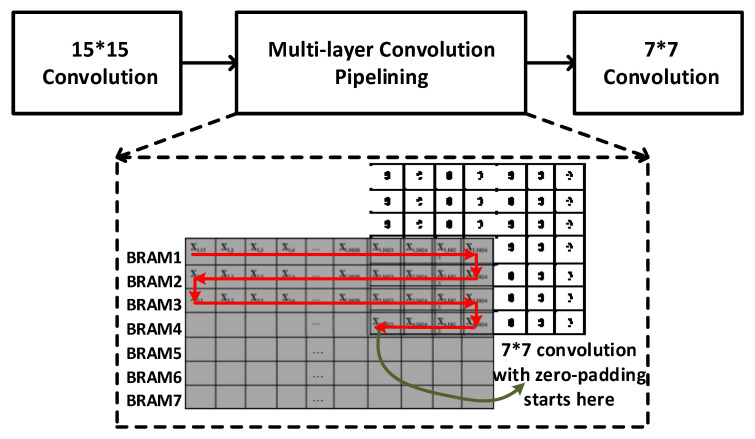
Multi-layer convolution pipelining.

To reduce the power consumption, a multi-layer convolution pipelining architecture is designed. As shown in Figure 5, once a few number of data are generated by the 15 × 15 convolution, the convolution of 7 × 7 can be started immediately. As zero-padding is involved in the convolution, for completing an output data for 7 × 7 convolution, instead of waiting for 7 new rows of data, only 3 rows plus 4 data is required.

This multi-layer convolution architecture significantly reduces the power consumption for data writing and reading. In addition, it also reduces the BRAM buffer size.

### 3.4. Convolution Filter Compression with Zero Skipping Convolution

In the biological retina inspired tone mapping algorithm, multiple convolution filters need to be read out repeatedly for the convolution. This causes large read time and power consumption. In addition, the storage of the convolution filters also consumes lots of BRAM resources.

**Figure 6:**
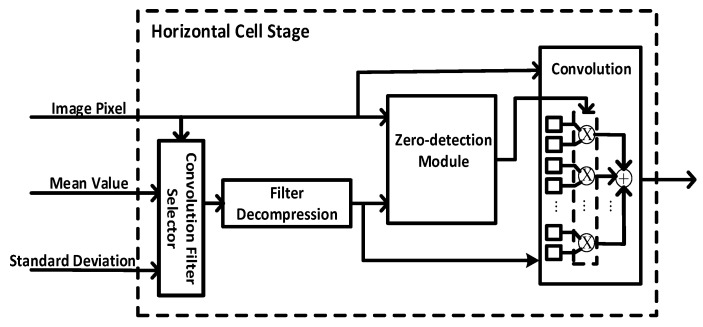
Convolution filter compression with zero skipping convolution.

We have observed the characteristics of the convolution filters and found that they are all symmetric. Moreover, some of the filters contain a lot of zero. Therefore, we have proposed to reduce the read time and power consumption by convolution filter compression with zero skipping convolution, as shown in Figure 6. Here there are two folds of compression.

Firstly, as the filters are symmetric with 4 lines (horizontal middle line, vertical middle line, and diagonal line), this feature can be used to compress the filters to 1/8. Secondly, the repeated consecutive data (e.g., a number of consecutive ‘1’ or ‘0’) is compressed by using run-length encoding. To further reduce the power consumption, a zero-detection module is designed to detect zero in the fetched filter data and skip the multiplication operation during convolution. By combining the convolution filter compression and zero skipping techniques, the power consumption as well as memory storage is largely reduced.

## 4. Experimental Results and Analysis

**Figure 7:**
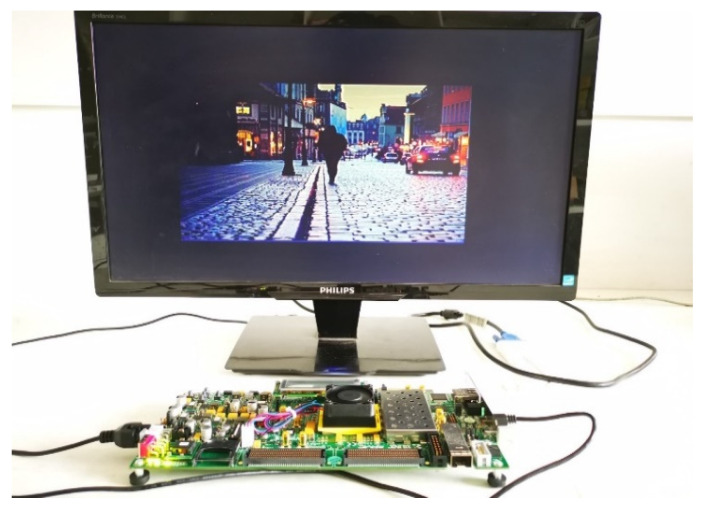
The experimental setup.

To evaluate and demonstrate the proposed biological retina inspired tone mapping processor, it has been implemented using a Xilinx Virtex 7 FPGA. Figure 7 shows the experimental setup. The input image is transferred from the computer to the FPGA. After processing, the processed image is sent to a monitor for displaying.

Several performance parameters have been evaluated (such as the peak signal to noise ratio (PSNR) [24], the structural similarity image index (SSIM) [24], clock frequency, throughput, and energy efficiency) and compared with several state-of-the-art tone mapping processors. Higher PSNR indicates smaller pixel error between the software and hardware, and higher SSIM indicates smaller structural error between the software and hardware. The proposed design shows higher PSNR and SSIM than other designs, as can be seen in Table 4 later.

**Figure 8:**
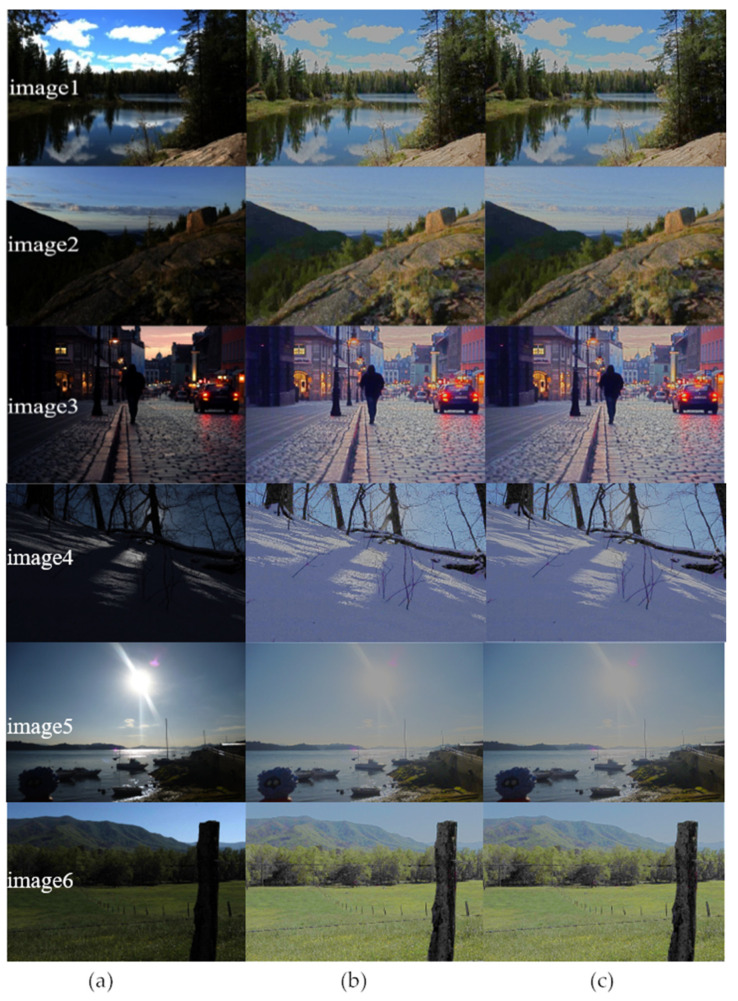
(**a**) Original image (**b**) Result of software processing (**c**) Result of hardware processing.

Figure 8 shows the images from large dataset of Mark Fairchild’s HDR Photographic Survey [25] before and after enhancement. Here, both software and hardware results are shown for comparison. It can be seen that after enhancement the details in dark areas are significantly improved. It can also be seen that the hardware results are almost the same as the software results. To further evaluate the quality of image after hardware processing, PSNR and SSIM have been calculated. The average PSNR and SSIM are 82.0661 dB and 0.9998, respectively, as shown in Table 2.

The maximum operating frequency of the processor is 150 MHz and the throughput is 189 frames per second for 1024 × 768 RGB image. The data width of the processor is 16-bit. Table 3 shows the hardware utilization of the design. We have also evaluated the power consumption using the Vivado power analysis tool based on post-layout simulation. The power consumption for processing 1024 × 768 RGB image is 819 mW and the calculated energy efficiency is 544,453 pixels/mW/s.

Table 4 compares the proposed tone mapping processor with several state-of-the-art designs. Among them [12] is the only biological retina inspired tone mapping processors that we found in the existing designs, while the others are non-biological retina inspired tone mapping processors. It can be seen that the proposed design has the highest PSNR and SSIM among the compared designs, which are 82.06 dB and 0.9999, respectively. Table 4 also shows the throughput and the energy efficiency in terms of pixels/mW/s. The higher the value, the higher the energy efficiency. With the multiple proposed design techniques to reduce the processing time and power consumption, the proposed design achieves a high throughput of 189 fps for processing 1024 × 768 image with a high energy efficiency of 544,453 pixels/mW/s, which outperforms other compared designs. The proposed biological retina inspired tone mapping processor is suitable for high-speed and energy-constrained image enhancement applications such as autonomous vehicle and drone monitoring.

## 5. Conclusions

In this work, a high throughput and energy-efficient retina inspired tone mapping processor is proposed for high-speed image enhancement on embedded devices. Several hardware design techniques have been proposed to improve throughput and energy efficiency, including data partition based parallel processing, adjacent frame feature sharing, multi-layer convolution pipelining, and convolution filter compression. Implemented on a Xilinx Virtex 7 FPGA, the proposed design achieves 189 frames per second for processing 1024 × 768 RGB images while consuming 819 mW, outperforming several state-of-the-art designs in terms of throughput and energy efficiency. This makes it suitable for high-speed and energy-efficient image enhancement applications.

## Figures and Tables

**Table 1:** Filter selection according to the value range of pixel.

Pixel Value Range	>m + 3s	(m + 2s, m + 3s)	(m + s, m + 2s)	(m − s, m + s)	(m − 2s, m − s)	(m − 3s, m − 2s)	<m − 3s
$\sigma_{n}\left(x,y\right)$	$\frac{sigma}{5}$	$\frac{2sigma}{5}$	$\frac{3sigma}{5}$	sigma	$\frac{3sigma}{5}$	$\frac{2sigma}{5}$	$\frac{sigma}{5}$

**Table 2:** Peak signal to noise ratio (PSNR) and structural similarity image index (SSIM).

Image	PSNR (dB)	SSIM
Image 1	80.8673	0.9999
Image 2	80.3091	0.9997
Image 3	83.2211	0.9999
Image 4	80.9242	1.0000
Image 5	82.7327	1.0000
Image 6	84.3424	1.0000
Average	82.0661	0.9999

**Table 3:** Hardware utilization.

FPGA Family	Clock Frequency (MHz)	Reg	LUT	DSP
Virtex7	150	22,693 (3.74%)	42,611 (14.04%)	675 (24.11%)

**Table 4:** Comparison with other tone mapping processors.

Ref.	FPGA Type	Retina-Inspired	Clock Frequency (MHz)	PSNR	SSIM	Throughput	Energy Efficiency (pixels/mW/s)
[1]	Cyclone III	NO	100	N/A	N/A	1024 × 768 126 fps	N/A
[11]	Cyclone III	NO	100	57.27 dB	0.9969	1024 × 768 126 fps	440,891
[12]	Spartan3	YES	40.25	30.00 dB	N/A	1024 × 768 60 fps	81,920
[13]	Virtex 6	NO	84.5	54.18 dB (MAX)	0.7050 (MAX)	1024 × 768 30 fps	66,459
[14]	Virtex 6	NO	69	30.00 dB	N/A	640 × 480 60 fps	61,645
Ours	Virtex 7	YES	150	82.06 dB	0.9999	1024 × 768 189 fps	544,453

## References

[1] Yang J., Hore A., Yadid-Pecht O. (2018). Local tone mapping algorithm and hardware implementation. Electron. Lett..

[2] Horé A., Yadid-Pecht O. A statistical derivation of an automatic tone mapping algorithm for wide dynamic range display. Proceedings of the 2014 IEEE International Conference on Acoustics, Speech and Signal Processing (ICASSP).

[3] Vonikakis V., Andreadis I., Gasteratos A. (2008). Fast centre-surround contrast modification. IET Image Process..

[4] Ferradans S., Bertalmio M., Provenzi E., Caselles V. (2011). An Analysis of Visual Adaptation and Contrast Perception for Tone Mapping. IEEE Trans. Pattern Anal. Mach. Intell..

[5] Jobson D.J., Rahman Z., Woodell G.A. (1997). A multiscale retinex for bridging the gap between color images and the human observation of scenes. IEEE Trans. Image Process..

[6] Rahman Z., Jobson D.J., Woodell G.A. Retinex processing for automatic image enhancement. Proceedings of the Human Vision and Electronic Imaging VII.

[7] Drago F., Martens W., Myszkowski K., Chiba N., Rogowitz B., Pappas T. Design of a Tone Mapping Operator for High Dynamic Range Images Based upon Psychophysical Evaluation and Preference Mapping. Proceedings of the Human Vision and Electronic Imaging VIII (HVEI-03).

[8] Zhang X.-S., Li Y.-J. (2016). A retina inspired model for high dynamic range image rendering. International Conference on Brain Inspired Cognitive Systems.

[9] Ofili C., Glozman S., Yadid-Pecht O. (2013). Hardware Implementation of an Automatic Rendering Tone Mapping Algorithm for a Wide Dynamic Range Display. J. Low Power Electron. Appl..

[10] Ofili C.A., Glozman S., Yadid-Pecht O. (2012). An in-depth analysis and image quality assessment of an exponent-based tone mapping algorithm. Int. J. Inf. Models Anal..

[11] Ambalathankandy P., Horé A., Yadid-Pecht O. (2019). An FPGA implementation of a tone mapping algorithm with a halo-reducing filter. J. Real-Time Image Process..

[12] Ureña R., Martínez-Cañada P., Gómez-López J.M., Morillas C., Pelayo F. (2012). Real-time tone mapping on GPU and FPGA. J. Image Video Proc..

[13] Leo Joseph L.M.I., Rajarajan S. (2019). Reconfigurable hybrid vision enhancement system using tone mapping and adaptive gamma correction algorithm for night surveillance robot. Multimed. Tools Appl..

[14] Martínez Cañada P., Morillas C., Ureña R., Gómez López J.M., Pelayo F.J. (2013). Embedded system for contrast enhancement in low-vision. J. Syst. Archit..

[15] Shahnovich U., Hore A., Yadid-Pecht O. Hardware implementation of a real-time tone mapping algorithm based on a mantissa-exponent representation. Proceedings of the 2016 IEEE International Symposium on Circuits and Systems (ISCAS).

[16] Popovic V., Pignat E., Leblebici Y. (2014). Performance Optimization and FPGA Implementation of Real-Time Tone Mapping. IEEE Trans. Circuits Syst. II Express Briefs.

[17] Drago F., Myszkowski K., Annen T., Chiba N. (2003). Adaptive Logarithmic Mapping for Displaying High Contrast Scenes. Comput. Graphics Forum.

[18] Eilertsen G., Mantiuk R.K., Unger J. (2017). A comparative review of tone-mapping algorithms for high dynamic range video. Comput. Graphics Forum.

[19] Devlin K. (2002). A Review of Tone Reproduction Techniques.

[20] Krasula L., Narwaria M., Fliegel K., Le Callet P. (2017). Preference of Experience in Image Tone-Mapping: Dataset and Framework for Objective Measures Comparison. IEEE J. Sel. Top. Signal Process..

[21] Zhuang L., Guan Y. Image Enhancement by Deep Learning Network Based on derived image and Retinex. Proceedings of the 2019 IEEE 3rd Advanced Information Management, Communicates, Electronic and Automation Control Conference (IMCEC).

[22] Steffens C., Drews P.L.J., Silva Botelho S. Deep Learning Based Exposure Correction for Image Exposure Correction with Application in Computer Vision for Robotics. Proceedings of the 2018 Latin American Robotic Symposium, 2018 Brazilian Symposium on Robotics (SBR) and 2018 Workshop on Robotics in Education (WRE).

[23] Ke X., Lin W., Chen G., Chen Q., Qi X., Ma J. EDLLIE-Net: Enhanced Deep Convolutional Networks for Low-Light Image Enhancement. Proceedings of the 2020 IEEE 5th International Conference on Image, Vision and Computing (ICIVC).

[24] Horé A., Ziou D. Image Quality Metrics: PSNR vs. SSIM. Proceedings of the 2010 20th International Conference on Pattern Recognition.

[25] Mark Fairchild’s HDR Photographic Survey. http://rit-mcsl.org/fairchild//HDR.html.

